# Essential chemistry for biochemists

**DOI:** 10.1042/EBC20160094

**Published:** 2017-09-26

**Authors:** Amanda L. Jonsson, Mark A.J. Roberts, J.L. Kiappes, Kathryn A. Scott

**Affiliations:** 1 Department of Chemistry, University of Wisconsin-Stevens Point, 2100 Main Street Stevens Point, WI 54481, U.S.A.; 2 Institute for Health Science Education, Queen Mary University of London, Barts and the London School of Medicine and Dentistry, Garrod Building, Turner Street, Whitechapel, London E1 2AD, U.K.; 3 Oxford Glycobiology Institute, Department of Biochemistry, University of Oxford, South Parks Road, Oxford, OX1 3QU, U.K.

**Keywords:** Non covalent interactions, Functional groups, Chemical reactions

## Abstract

Within every living organism, countless reactions occur every second. These reactions typically occur more rapidly and with greater efficiency than would be possible under the same conditions in the chemical laboratory, and while using only the subset of elements that are readily available in nature. Despite these apparent differences between life and the laboratory, biological reactions are governed by the same rules as any other chemical reaction. Thus, a firm understanding of the fundamentals of chemistry is invaluable in biochemistry. There are entire textbooks devoted to the application of chemical principles in biological systems and so it is not possible to cover all of the relevant topics in depth in this short article. The aim is instead to provide a brief overview of those areas in chemistry that are most relevant to biochemistry. We summarize the basic principles, give examples of how these principles are applied in biological systems and suggest further reading on individual topics.

## The elements of biochemistry

Biochemical systems carry out an enormous variety of chemical reactions with great efficiency. These reactions can be catabolic, breaking down larger molecules to release energy and to generate precursors for further reactions, and/or anabolic, combining molecules together to generate biologically useful molecules. When a chemist carries out a reaction in the laboratory, they have numerous different techniques that can be applied to increase the yield or rate of a reaction; they can alter the temperature or pressure or perhaps add a catalyst. In contrast, biological systems have to carry out the reaction at the temperature maintained by the organism, and, with the exception of organisms living deep in the ocean, at atmospheric pressure. In biological systems, enzymes are employed to increase the rate of reaction; enzymes are proteins whose substrate-binding site acts to lower the energy of high energy species along the reaction pathway from starting material to product. They can achieve enormous enhancement in the rate of reaction compared with the uncatalysed reaction. The classic example is the enzyme triose phosphate isomerase which interconverts dihydroxyacetone phosphate and d-glyceraldehyde-3-phosphate during the breakdown of glucose. This reaction occurs 10^9^ times faster in the presence of the enzyme than when uncatalysed [1].

**Figure 1:**
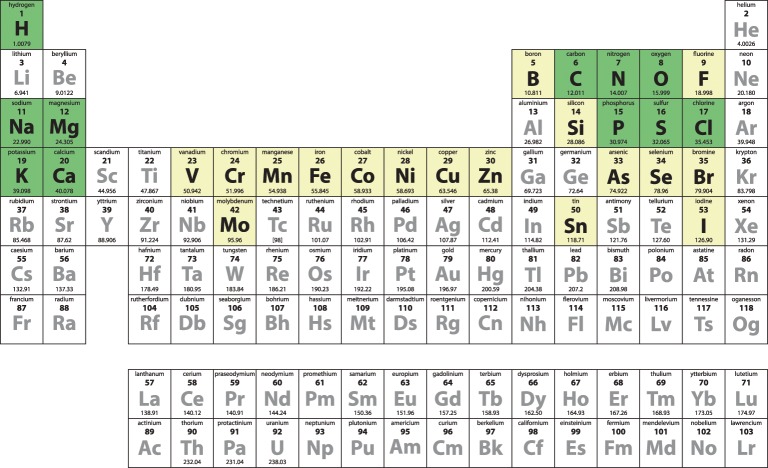
Periodic table illustrating the elements essential for life The 28 elements essential for animal life are indicated by coloured squares; trace elements are shown in yellow and those present in larger quantities are shown in green. The six most abundant elements in the human body are carbon, hydrogen, oxygen, nitrogen, phosphorus and calcium, accounting for almost 99% of the mass of an adult human. Carbon, hydrogen, oxygen and nitrogen are the building blocks of organic biomolecules, calcium is present in large amounts in bones and teeth (in addition to being vital for cell signalling in smaller amounts), phosphorus is likewise found in bones and teeth (smaller quantities are a vital part of DNA, adenoside triphosphate – the energy currency of the cell – and play an important role in cell signalling).

These rate enhancements are particularly remarkable when we consider that less than a third of the naturally occurring elements are used by biological systems. In order to be exploited in a biological system, elements must be sufficiently abundant in a form that can be taken up by living things. Thus, many catalytic species that are in common use in the laboratory are simply not available for biochemical reactions, for example palladium, used in the addition of hydrogen across a double bond. Of the elements in the periodic table, 28 are essential for animal life (Figure 1); the most recent element found to be essential is bromine, which was found to be required for the proper formation of networks by the protein collagen IV in 2014 [2]. Of the 28 essential elements, 11 make up 99.9% of the atoms in the human body. The other 17 are known as trace elements and are present in very small amounts, ranging from a milligram to gram quantities in an adult human.

## Atomic structure and bonding

Electrons in atoms are organized into a series of shells with successively higher energies (and greater distance from the nucleus). The shells are identified by the principal quantum number which takes integer values from one to seven for the elements of the current periodic table. The shell with principle quantum number 1 has the lowest energy with 2 being the next highest in energy, and so on. Within a shell there are subshells designated by the letters s, p, d and f, and within each subshell electrons occupy orbitals: regions of space that may be occupied by up to two electrons, and whose energy and shape can be described mathematically by an equation known as the wave function. It is important not to confuse orbitals with an orbit; electrons do not move around the nucleus along a fixed path as they would in an orbit. Instead the wave function allows us to calculate the probability of finding an electron at a particular position around the nucleus. Different subshells have different numbers of orbitals; s subshells have just one orbital and can therefore accommodate only two electrons, p subshells have three orbitals which can be filled by six electrons in total, and d subshells have five orbitals accommodating up to ten electrons. Shells are filled according to the Aufbau principle, i.e. filling the shell with the lowest principal quantum number first.

Arrangements of electrons or *electron configurations*, in which the outer shell (the occupied shell with highest principle quantum number) is full are more favourable than those in which it is partially filled. Of the elements in the periodic table, only the noble gases in group 18 have full outer shells. Bond formation, the movement of electrons between atoms, allows other elements to achieve this configuration. Chemical bonds can be covalent, where electrons are shared between atoms or ionic, where electrons are transferred from one atom to another resulting in one positively and one negatively charged species. Looking at the number of electrons in the outer or valence shell enables us to work out how many bonds an atom would need to form in order to fill its outer shell. It is important to note, however, that a bond will only actually form if the energy of the electrons in the bond is lower than the energy of those electrons in the isolated atoms.

As the majority of biological chemistry relates to covalently bonded molecules composed primarily of the elements carbon, hydrogen, nitrogen and oxygen, it is particularly important to know how many bonds each of these elements form. Hydrogen, with its single valence electron requires one additional electron to achieve the noble gas configuration and therefore makes only one bond. H^+^ ions (often just called protons), in which the hydrogen atom has lost an electron, also play many roles in biological systems – for example the formation of ATP is driven by a concentration gradient of H^+^ ions across a membrane. Carbon, with four valence electrons, achieves a full outer shell by forming four covalent bonds, for example by sharing an electron with each of four other atoms. Nitrogen has five valence electrons and forms three covalent bonds leaving one pair of non-bonded electrons; a lone pair. The lone pair is important for the reactivity of nitrogen as it can be used to make a new bond with electron-poor species in chemical reactions. The lone pair can also be shared with an H^+^ ion leading to the formation of ammonium, i.e. the lone pair allows nitrogen to act as a base. Finally, oxygen with six electrons makes two covalent bonds and has two lone pairs of electrons. As with nitrogen, the presence of lone pairs on oxygen makes this atom reactive towards electron-poor species, including H^+^. Although we often refer to H^+^ ions as though they exist in that form, in water free H^+^ forms the hydronium ion H_3_O^+^ very rapidly.

**Figure 2:**
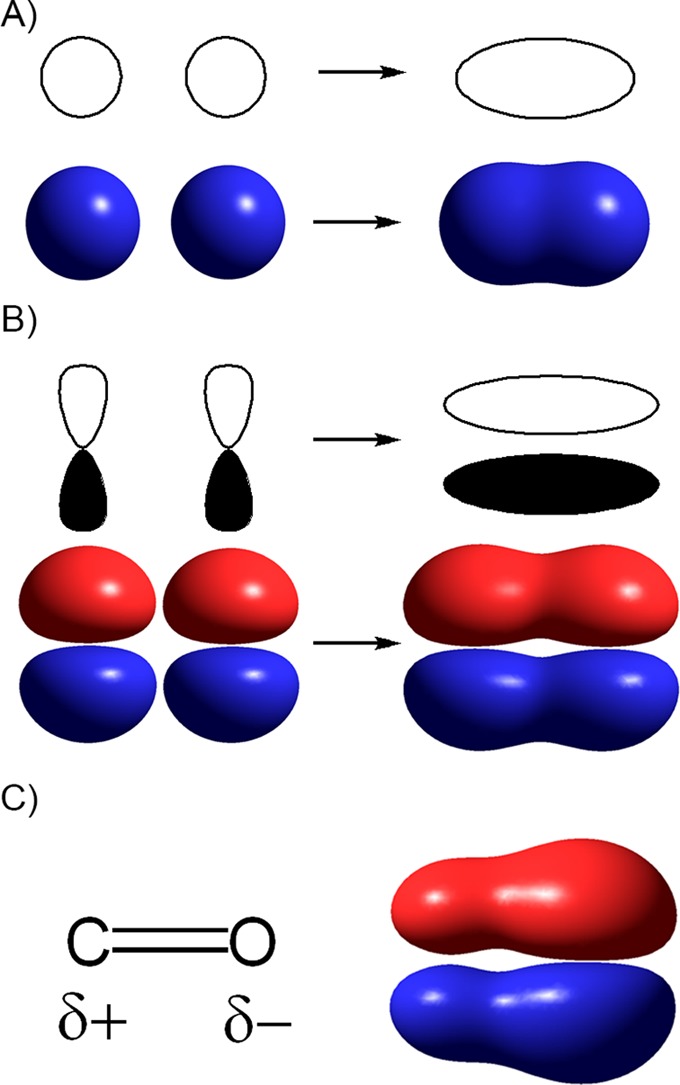
σ and π bonds In these images, atomic orbitals and bonds are depicted as line drawings indicating shape and as isosurfaces, regions of space enclosing a defined fraction of the electron density. (**A**) When two s-orbitals overlap, a σ-bond is formed. In σ bonds, the atomic orbitals overlap ‘head on’ and there is electron density between the two nuclei in the bond. As a result, these bonds are typically strong compared with π bonds between the same elements. (**B**) In a π bond, two p-orbitals overlap laterally, and the nuclei lie in a plane in which there is no electron density. There is less overlapping between orbitals in a π bond than in a σ bond and so these bonds are typically weaker. It should be noted that a double bond, consisting of a σ and π bond is stronger than a single bond between the same two elements as the strength of both the σ and π components are included in the bond strength. (**C**) When a bond is formed between carbon and oxygen, there will be more electron density located near the oxygen atom, as illustrated here in the isosurface for a carbon–oxygen π bond.

When an ionic bond is formed, electrons are transferred completely from one atom to the other. The interaction in an ionic bond is entirely Coulombic in nature (i.e. only due to the force of attraction between positive and negative particles) and there is no directionality to the interaction. Such a bond occurs when the elements differ widely in their ability to attract electrons or more formally, when there is a very large energy difference between the valence orbitals (the outermost orbitals containing the electrons available to participate in bonding) in the two atoms. Covalent bonding involves sharing of electrons between atoms and occurs when the two atoms are more similar in their ability to attract electrons; i.e. when the valence orbitals have similar energy. Sharing of electrons in a covalent bond requires atomic orbitals on each of the atoms to interact with each other. One of the consequences of this is that, in contrast with ionic bonds, covalent bonds are directional: a new bond cannot form in a region of space where the orbitals that interact to form the new bond have no electron density. Two classes of covalent bonds occur commonly in biological chemistry, σ bonds and π bonds (Figure 2A,B). Although a pair of electrons are shared when a covalent bond is formed, however, this sharing is not necessarily equal. Atoms with a strong ability to attract electrons, i.e. low energy valence orbitals, are referred to as electronegative. If the two atoms forming a covalent bond differ in electronegativity then there will be greater electron density closer to the more electronegative atom. This results in a permanent dipole, with one atom partially negatively charged and the other partially positively charged (Figure 2C).

### Delocalization of electrons

The description of covalent bonding has so far assumed that a covalent bond involves the sharing of one pair of electrons between two atoms, i.e. that the bond is localized. For the majority of biological molecules this description is adequate, however in some cases this description of bonding does not explain the observed properties of a molecule.

**Figure 3:**
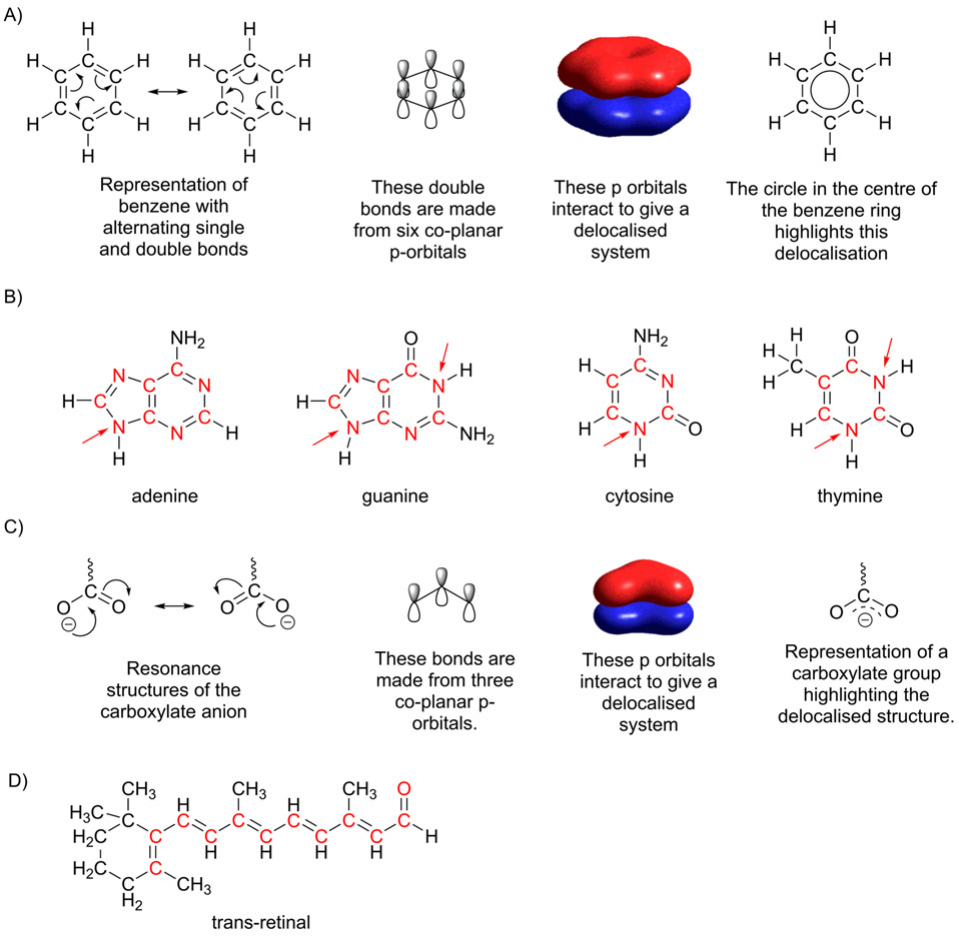
Delocalized structures (**A**) Each of the six carbon atoms in benzene contribute a p-orbital to the delocalized system. A circle in the centre of the ring can be used to highlight the fact that the system is delocalized, however many biochemists prefer to use the alternating bond representation. (**B**) The four bases found in DNA, all have delocalized rings of p-orbitals; in this diagram atoms shown in red each contribute a p-orbital to the delocalized system. It is not immediately obvious that the atoms indicated with an arrow can contribute a p-orbital, however using a more sophisticated approach to bonding we can show that this is the case. Adenine and guanine each have ten electrons in a delocalized ring, while cytosine and thymine have six. (**C**) Representation of the delocalized carboxylate anion. In this system, four electrons two from the double bond and two from the negatively charged oxygen are delocalized over three atoms. (**D**) Retinal has a linear delocalized system including 12 p-orbitals. Each of the atoms that contributes a p-orbital to the delocalized system is shown in red.

A well-known example is benzene. It was originally thought that benzene contained three alternating single and double bonds, however measurements showed that the bonds were all of equal length. We now consider the bonding in benzene not as three pairs of p-orbitals each interacting to make one double bond, but six p-orbitals each interacting with its neighbours to create a ring of electron density above and below the plane of the carbon atoms (Figure 3A). Compounds with delocalized rings of electrons are of major importance in biological systems. For example, the bases in DNA all contain delocalized rings as a part of their structures and favourable interactions between the electrons in these rings (referred to as π–π stacking) contribute to the stability of the DNA double helix (Figure 3B).

Delocalization of electrons does not only occur in rings; another type of system where delocalization occurs is where three (or more) parallel p-orbitals are adjacent. Consider the carboxylate anion (discussed in *The carbonyl functional group* section) where a carbon atom makes a double bond with one oxygen atom and a single bond with a negatively charged oxygen atom. In this structure, we can visualize the negative charge on the oxygen being used to make a new double bond with the carbon atom and the existing double bond breaking to leave a negative charge on the oxygen atom (Figure 3C). Although we can visualize single and double bonds exchanging position within the molecule, this is not an accurate description of bonding in the molecule. In reality, the electron density is spread over three p-orbitals, and a higher electron density exists on the two oxygen atoms than on the central carbon atom. Delocalization of electron density across three p-orbitals is also important in explaining why the bond formed between two amino acids in a protein chain is planar (see *Functional groups found in amino acids* section). Molecules with electrons are delocalized over a larger number of adjacent parallel p-orbitals are also common in biology. These molecules are usually referred to as conjugated and can be identified by their alternating chain of single and double bonds. For example, retinal, the light-absorbing molecule that is bound to the protein opsin in the photoreceptor cells responsible for vision in mammals, has electron density delocalized across 12 p-orbitals (Figure 3D). The long delocalized system is essential for the absorption of light in the visible region of the electromagnetic spectrum.

### Non-covalent interactions

Non-covalent interactions, such as the π–π stacking mentioned in the above section, arise due to electrostatic interactions between two different molecules or within the same molecule between atoms that are not bonded together, without the sharing of electrons via a covalent bond. These interactions are much weaker than the covalent bond but they occur very frequently and, as a result, can have a huge influence on the properties of a molecule. Many biomolecules are macromolecules with thousands of atoms and therefore make many hundreds of thousands of non-covalent interactions. Non-covalent interactions are particularly important in proteins. Proteins are polymers of amino acids, synthesized in a linear chain on the ribosome. Each protein chain folds into a specific 3D structure that is essential for its function; non-covalent interactions between the constituent amino acids determine the 3D structure. Non-covalent interactions are also important in DNA where they help to ensure that the sequence of DNA is preserved upon replication; in the lipid bilayer where non-covalent interactions between lipids create a barrier around the cell; and in molecular recognition (discussed in more detail in [3]). There are several classes of non-covalent interactions; here we discuss van der Waals interactions, hydrogen bonds and briefly, ionic interactions. We also discuss the ‘hydrophobic effect’ which is commonly invoked to explain why non-polar molecules do not disperse in water and why proteins fold.

#### van der Waals interactions

A dipole is an uneven distribution of electron density within a molecule such that one region of the molecule has a higher electron density than the other and the two regions are equally but oppositely charged. van der Waals interactions occur when a dipole on one molecule interacts with the dipole on another molecule. These dipoles can be permanent or instantaneous. Permanent dipoles occur due to the uneven charge distribution in a covalent bond between two elements that differ greatly in electronegativity. The partially positively charged (δ^+^) atom on one molecule can interact favourably with the partially negatively charged (δ^–^) atom on another. Interactions between instantaneous dipoles are called London dispersion forces. They are the weakest among the non-covalent interactions, but also the most prevalent. London dispersion forces occur because the electron density in an atom or molecule does not have an even distribution; at any one time the electron density may be higher in one region than the other. The electron density is redistributed with time, thus the regions of high electron density are different from one moment to the next. The uneven charge distribution is called *instantaneous dipole*. The distribution of the electron density in neighbouring molecules is influenced by the dipole of the first molecule; areas of relative high electron density on one molecule induce an area of low electron density on the neighbouring molecule and vice versa; thus neighbouring molecules form instantaneous dipoles that attract each other. Typically, each of these interactions has a strength of only ∼2 kJ mol^−1^ (compared with covalent bonds which typically have a strength of hundreds of kJ mol^−1^) and the magnitude of the interaction is strongly distance dependent, approaching zero at a separation of ∼8–10 Å. When molecules have large surface areas that can come into close contact, for example in biological macromolecules, these interactions can make a huge contribution to the total free energy.

#### Hydrogen bonding

Hydrogen bonds are a special case of dipole–dipole interaction, but are considered separately here as they are vital for the function of many biochemical systems. A covalent bond between hydrogen and an electronegative atom, such as oxygen, nitrogen or fluorine, is polar, with electron density in the vicinity of the hydrogen much lower than that around its bonding partner. The favourable interaction between the δ^+^ hydrogen of one X–H bond (where X is an electronegative element) and the δ^–^ X atom of another is called as a hydrogen bond. Hydrogen bonds are stronger than van der Waals interactions, but weaker than covalent bonds, with a typical strength between 5 and 40 kJ mol^−1^. It is important to note that the strength of a hydrogen bond depends heavily on the geometry of the atoms involved; the bonds are strongest when the three atoms involved in the bond are collinear. Hydrogen bonds are responsible for the specificity of base pairing, A to T and C to G, in DNA strands. They also play a key role in formation of structural elements during protein folding.

#### Ionic interactions

Ionic interactions occur between species that have full, permanent charges, i.e. ions. These interactions are much stronger than hydrogen bonds and London dispersion forces, but are much less common in biological systems. Ionic interactions between oppositely charged amino acids play an important role in stabilizing protein structure, as demonstrated by changes in protein shape with pH. Proteins have evolved to form the correct 3D structure at the pH of the environment where they function. At pH values far above and below the physiological range the charges of some of the amino acids forming the protein are changed (see section on *pH and pK*_a_ for details). This changes the ionic interactions within the protein, in many cases causing the protein to unfold. Ionic interactions are particularly important in stabilizing proteins found in thermophilic organisms – those that thrive at temperatures above 40°C. Ionic interactions also play a key role in the binding of charged molecules such as ATP to their macromolecular partners.

#### The ‘hydrophobic effect’

**Figure 4:**
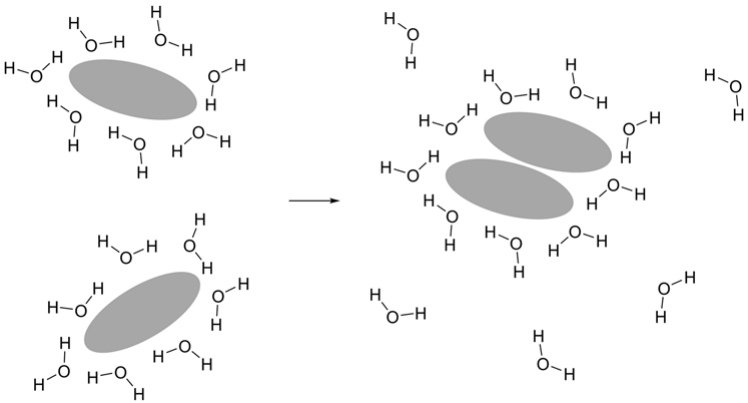
The hydrophobic effect Water molecules form an ordered structure around a hydrophobic molecule (shown in grey). When two hydrophobic molecules aggregate the surface area exposed to water is reduced; this reduces the number of ordered water molecules in the hydration shell. Having more water molecules disordered in solution is entropically favourable.

The ‘hydrophobic effect’ is not a separate class of non-covalent interaction, but it is discussed here as the factors that give rise to this effect are very important for structure formation by biological macromolecules and especially proteins. Many molecules or parts of molecules, are hydrophobic or ‘water-hating’ and tend to cluster together when placed in water; this behaviour is often referred to as the hydrophobic effect. The hydrophobic effect is thought to be primarily entropic (see *Why do chemical reactions happen?* section), arising due to changes in hydrogen bonding within water in the presence of a non-polar molecule. Water forms an extensive network of transient hydrogen bonds which are broken and formed as the water molecules move. If a non-polar species is placed in water, it disrupts the hydrogen bonding networks. To minimize the number of hydrogen bonds lost due to this disruption water forms an ordered shell around the non-polar species. To make a large number of these ordered shells is entropically very unfavourable; clustering non-polar molecules together minimizes the non-polar surface area exposed to water and hence also minimizes the number of ordered water molecules (Figure 4). This is believed to be the primary driving force for burying non-polar groups on the inside of globular proteins and arranging polar groups on the outside. It is also the driving force for the self-assembly of lipid bilayers (discussed in detail in [4]).

## Functional groups in biochemistry

**Figure 5:**
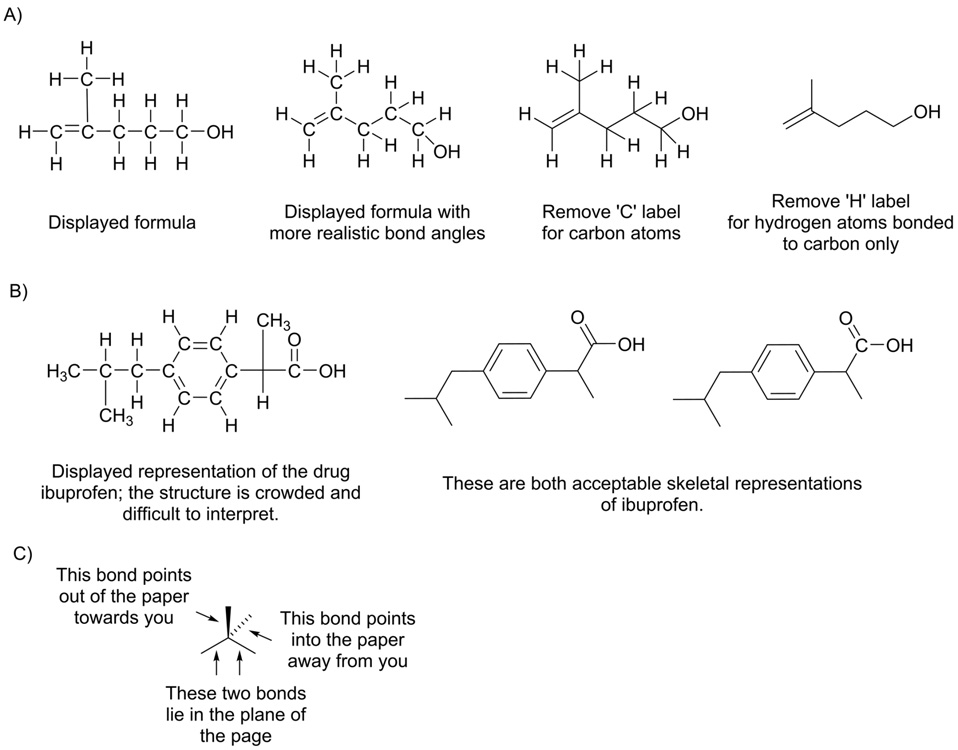
Displaying molecules using a skeletal representation The skeletal representation makes use of the fact that carbon and hydrogen are the most common elements in organic compounds, and also that carbon almost always forms four covalent bonds. (**A**) The process of converting a displayed formula into a skeletal formula has three stages: (i) the molecule is drawn with the carbon chain ‘staggered’ in a zigzag; this is important to allow correct identification of the number of carbon atoms in the final molecule and is a better representation of the actual bond angles. (ii) The ‘C’ label for carbon atoms is omitted. (iii) The ‘H’ label for hydrogen atoms is omitted only for hydrogen atoms bonded to carbon. When interpreting a skeletal formula, we assume that carbon will make four bonds; if a carbon appears to make fewer than four bonds in the skeletal representation, then the ‘missing’ bonds are assumed to be to hydrogen atoms. Using the skeletal representation highlights the heteroatoms present in the molecule and becomes particularly useful once we consider how to display different isomers. (**B**) The guidelines for drawing skeletal structures are relatively flexible and in some cases, for example where we wish to draw attention to a particular group of atoms, the ‘C’ symbol for some of the carbon atoms may be shown. It is important not to omit any hydrogen atoms bonded to carbon atoms that are explicitly displayed in this way. (**C**) Wedge and hashed bonds can be used to illustrate the direction of bond vectors relative to the plane of the page.

**Figure 6:**
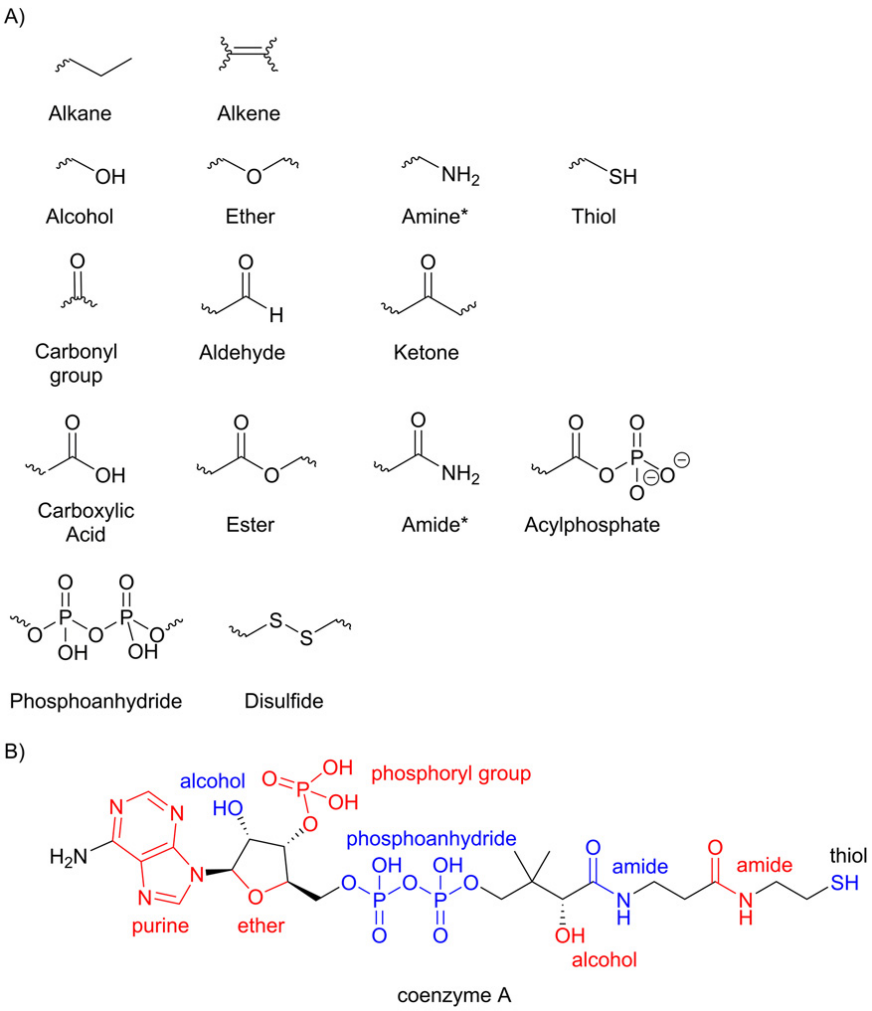
Functional groups (**A**) This figure illustrates the some of the functional groups that occur frequently in biological systems. In these structures, the wavy bonds represent connections to the rest of the molecule. *In both amine and amide, one or both of the hydrogen atoms bonded to the nitrogen may be replaced by a carbon atom. (**B**) Many biological molecules include a large number of different functional groups, as illustrated by the structure of coenzyme A with the functional groups labelled in blue and red.

The chemical reactivity of an organic compound depends upon the way in which its atoms are bonded together. Certain collections of atoms having the same connectivity occur frequently in organic compounds and these collections are called functional groups. Functional groups have a characteristic chemical behaviour, and it is possible to predict some of the properties of a molecule based on which functional groups are present. When discussing organic compounds we will use the skeletal representation; those unfamiliar with this representation should refer to Figure 5. Functional groups that are commonly found in biological molecules are shown in Figure 6.

### Hydrocarbon functional groups

Functional groups containing only carbon and hydrogen may be saturated (containing only carbon–carbon single bonds) or unsaturated (containing one or more carbon–carbon double or triple bonds). Alkanes are saturated molecules and are chemically unreactive compared with other functional groups. Alkanes are non-polar and hydrophobic, interacting less favourably with water than with other non-polar molecules. Alkenes are unsaturated hydrocarbons and they are also hydrophobic. They are, however, more reactive than alkanes, in particular towards the addition of atoms across the double bond. Alkanes and alkenes are abundant in cell membranes, where their hydrophobic nature is important in creating a barrier around the cell that is impermeable to molecules including water, ions and other polar molecules.

### Functional groups in which carbon forms a single bond with an electronegative atom

The alcohol, ether, amine and thiol (–SH) functional groups all contain a carbon atom forming a single bond with an electronegative heteroatom (any atom that is not carbon or hydrogen). The difference in electronegativity between the carbon and the heteroatom makes these bonds polar; there is a higher probability of finding the electrons in the bond close to the electronegative atom than to the carbon atom. To indicate this polarization, we often show carbon with a partial positive charge and the electronegative atom with a partial negative charge. This uneven charge distribution greatly influences the reactivity of these molecules, with the carbon atom potentially being reactive towards electron-rich species known as nucleophiles. The electronegative atoms themselves may also be reactive. Oxygen, nitrogen and sulphur all have lone pairs of electrons that can form new covalent bonds in a chemical reaction; amines in particular are reactive towards protons, forming –NH_3_^+^ at physiological pH (see *pH and pK*_a_ section). The uneven charge distribution also influences the way these molecules interact with water; these functional groups form favourable van der Waals dipole–dipole interactions with water molecules, as described earlier, and their presence will increase the solubility of a compound.

### The carbonyl functional group

The carbonyl group, a carbon atom forming a double bond with an oxygen atom, is a part of many different functional groups. Carbonyl groups are found in a vast range of biological molecules, including proteins, DNA and sugars and they take part in many biological reactions. The reactivity of the carbonyl group varies depending upon which other atoms are bonded to the carbonyl carbon. The simplest carbonyl compounds are aldehydes, which have at least one hydrogen atom bonded to the carbonyl carbon, and ketones in which the carbonyl carbon is bonded to two other carbon atoms. As is the case with alcohols, the carbon–oxygen double bond in carbonyl groups is polarized, with the carbon atom partially positively charged and the oxygen atom partially negatively charged. The carbonyl carbon is particularly susceptible to attack by nucleophiles and this makes the carbonyl group useful for the formation of new carbon–carbon bonds. One important example where carbonyl chemistry plays a key role is the formation of the *ketone bodies* (acetoacetate, β-hydroxybutyrate and the breakdown product acetone). Ketone bodies are water-soluble species that can act as an alternative energy source to glucose in some tissues when glucose is scarce, for example during starvation or intense exercise. The ketone bodies are formed in the liver and then released into the bloodstream for use as a fuel in the heart, brain and muscle.

Carboxylic acids, esters, amides and acyl phosphates are known as carbonyl derivatives. As the name suggests, carboxylic acid functional groups readily lose a proton (the proton bonded to the oxygen atom); the resulting species is referred to as a carboxylate group or carboxylate anion. In these functional groups, the carbonyl carbon forms an additional single bond with an electronegative element. Although at first glance, it might seem as though this should make these carbonyl compounds more susceptible to nucleophilic attack than aldehydes or ketones (because the carbonyl carbon is bonded to more electronegative atoms) this is not actually the case due to delocalization of electrons within the functional group, as described earlier (see section: *Delocalization of electrons*). In biological chemistry, reactions involving the interconversion of carbonyl derivatives are very common, for example in protein synthesis where carboxylic acid groups are converted into amides.

### Other functional groups

The phosphoanhydride and disulphide groups are much more common in biochemical systems than in the organic chemistry laboratory. Phosphoanhydride groups are found between phosphate groups in ATP, and attack on this functional group by water or other electron-rich species is important in driving chemical reactions forward in the cell. Disulphide linkages can be formed from two thiol groups under reducing conditions. They play an important role in the function of the cellular antioxidant glutathione and also in stabilizing the structure of extracellular proteins.

### Functional groups found in amino acids

**Figure 7:**
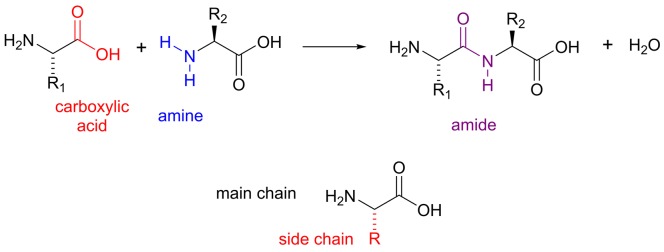
Amide bonds in a polypeptide In this figure, the symbol R is used to denote the different functional groups that distinguish the amino acids. The amino acids that polymerize to form a polypeptide or protein are joined together through amide bonds. This bond is often referred to as a peptide bond. The carbon, oxygen and nitrogen atoms of the amide group all lie in the same plane with the lone pair on the nitrogen forming a delocalized system with the electrons from the carbon–oxygen double bond, as was described for the carboxylate anion. The planarity of this bond has important implications for protein structure.

**Figure 8:**
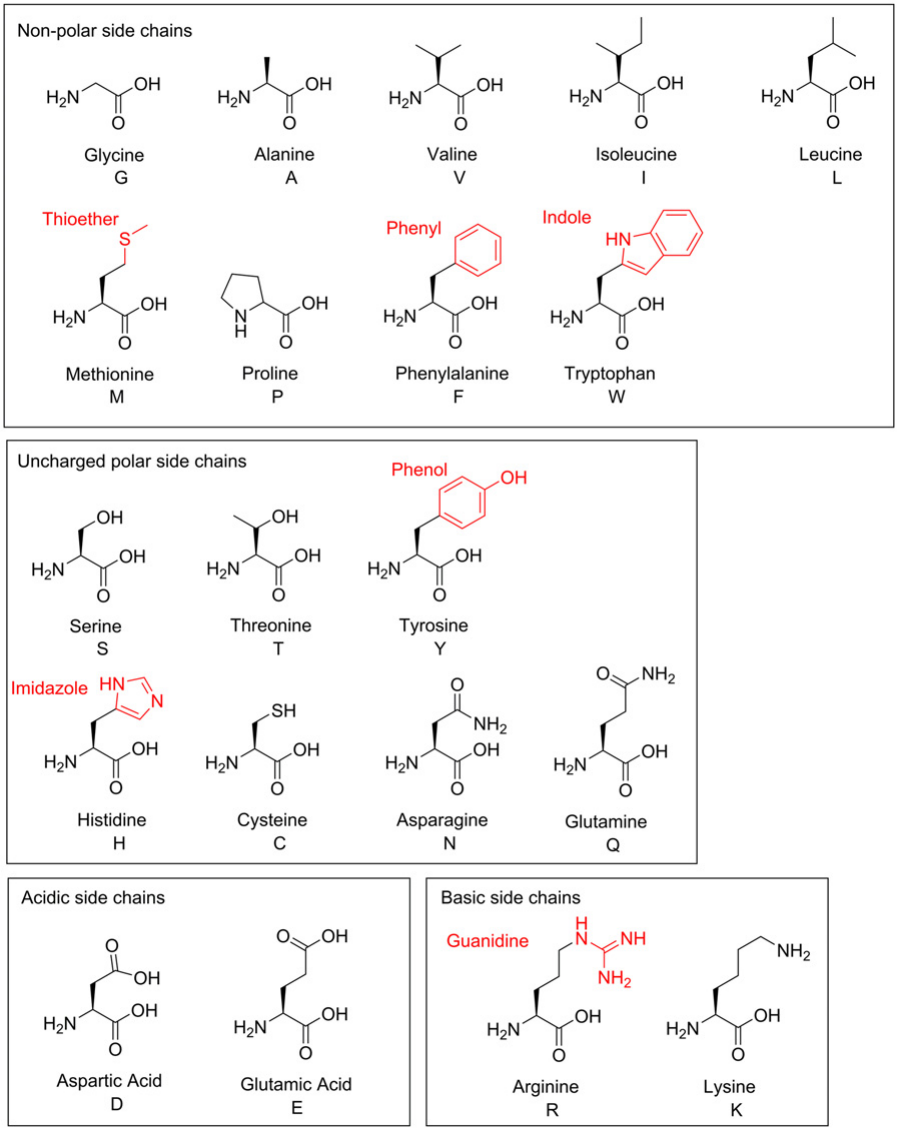
Amino acids The twenty amino acids commonly found in proteins. Functional groups not illustrated elsewhere are labelled in red. Full names and one-letter codes are given. Amino acids are categorized as non-polar, uncharged polar, acidic and basic according to the composition of their side chains. Methionine and tryptophan are classified as non-polar, despite including the polar atoms sulphur and nitrogen respectively, as both are typically found in the interior of a protein. The sulphur atom in methionine does not participate in hydrogen bonds or act as a nucleophile in enzymatic reactions. In tryptophan, the nitrogen lone pair is delocalized through the indole ring, ensuring that it is likewise unavailable as a nucleophile. Side chains which frequently form hydrogen bonds are classified as polar, those that lose a proton at physiological pH are acidic, and those that gain a proton are basic. Histidine could be considered both polar uncharged and basic as it exists in both neutral and positively charged state at physiological pH.

Amino acids are named for the two functional groups present in their shared molecular backbone, the amine and carboxylic acid groups. Amino acids are the building blocks of proteins/polypeptides with an amide functional group being formed from the amine and carboxylic acid groups during protein synthesis. By convention, the groups that occur in all amino acids are called the ‘main chain’ or ‘backbone’, while those that differ between amino acids are called the ‘side chains’ or ‘R group’ (Figure 7). Twenty amino acids commonly occur in natural proteins. These are shown in Figure 8, along with the corresponding one-letter codes that are used as an abbreviation when describing the order in which amino acids appear in a protein. Amino acid side chains include a wide variety of different functional groups, from alkanes in alanine, valine, isoleucine and leucine, to the imidazole group in histidine and guanidine group in arginine. The polypeptide chain of a protein folds up into a specific 3D structure, determined by the order in which the amino acids are added to the chain. It can be useful to categorize amino acids according to the chemical properties of their side chains since these determine the role that a particular type of amino acid most commonly plays in protein structure and function. Here, we have classified side chains as non-polar, polar but uncharged, acidic and basic, but it is important to note that several amino acids could be placed in more than one category. In general, amino acids with non-polar side chains are predominantly found in the interior of a protein. Two key exceptions are glycine and proline. Although both lack polar atoms in their side chains, the unusual ring structure of proline and the small size of glycine make them particularly useful in forming loops and so they are often found on the protein surface. The polar side chains make hydrogen bonds either with water or with other polar amino acids and are commonly found on the surface of a protein or buried as a part of an enzyme active site. The polar side chains with alcohol functional groups (serine, threonine and tyrosine) play a particularly important role on the surface of many proteins involved in cell signalling, where they can be modified by the addition of a phosphate group. The addition of the phosphate group changes the chemical nature of the protein surface from polar to charged and therefore alters the interaction with partner proteins. The amino acid cysteine is important in stabilizing the structure of many extracellular proteins through formation of a *disulphide* bond, two cysteine side chains linked together through their sulphur atoms (Figure 6). The acidic side chains lose a proton to become negatively charged at physiological pH, while the basic side chains gain a proton to become positively charged (see section *pH and pK*_a_ for more details). Charged side chains are often found at the surface of proteins in pairs with oppositely charged side chains where the resulting ionic interaction helps to stabilize the protein structure.

## Isomerism

Isomers have the same molecular formula, but differ in structure. Isomers are classified according to whether they differ in atom connectivity or in 3D shape.

### Structural isomerism

**Figure 9:**
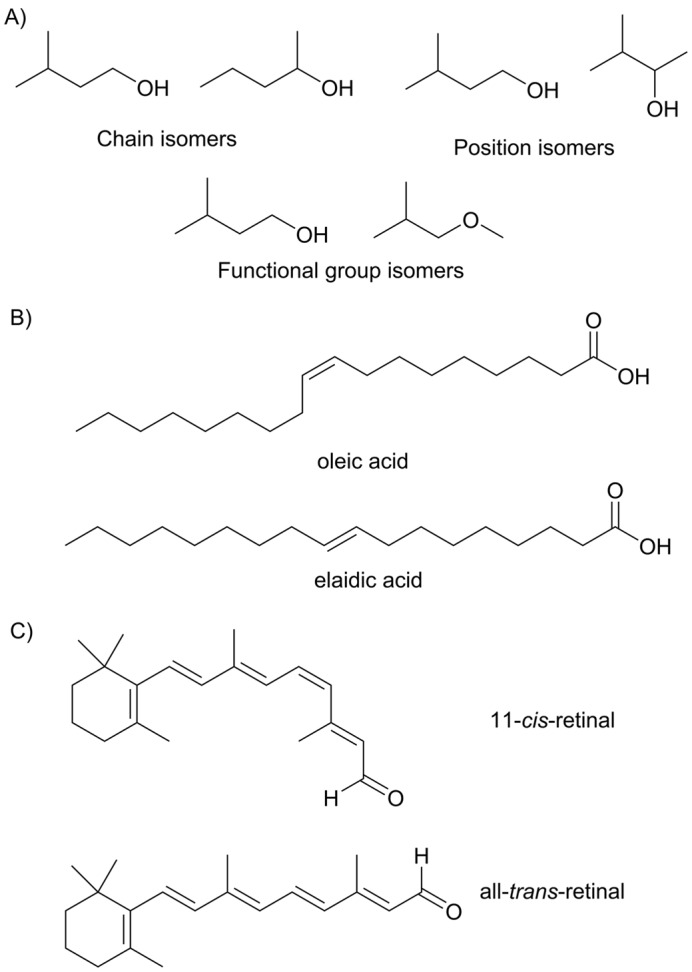
Structural isomers and *cis-trans* isomers (**A**) This figure illustrates the three classes of structural isomers: chain isomers, position isomers and functional group isomers. (**B**) The fatty acid oleic acid has a *cis*-double bond and is often incorporated into the lipids forming the cell membrane. In contrast, elaidic acid which has a *trans*-double bond is not found in cell membranes. (**C**) 11-*cis*-retinal is converted into all-*trans*-retinal on absorption of light of the appropriate energy in the first step of light detection in the eye.

Structural isomers occur when the atoms are bonded together in a different order. Structural isomers are classified into three groups: chain isomers, position isomers and functional group isomers. Chain isomers have the same functional groups bonded to different carbon frameworks. Position isomers have the same carbon framework and type of functional groups but differ in the position of those functional groups. Functional group isomers contain different functional groups (and hence typically have different carbon frameworks) (Figure 9A).

### Stereoisomerism

In biochemistry, a more subtle kind of isomerism, stereoisomerism, is vitally important. Here the connectivity of the atoms in the isomers is identical, but the spatial arrangement of the atoms differs. The two classes of stereoisomers we will consider here are *cis-trans* isomers and molecules with chiral centres.

#### *Cis-trans* isomerism

*Cis-trans* isomerism, sometimes known as geometrical isomerism, occurs in molecules with double bonds and arises because there is no rotation of groups about a double bond. In the *cis*-isomer, the extending carbon chains are on the same side of double bond, while in the *trans*-isomer they are on opposite sides of the bond. The two different isomers have different physical and chemical properties; for example melting points often differ considerably between the *cis* and *trans* isomers.

Having the correct *cis* or *trans* isomer of a molecule can be vital for biological function, for example in the lipid membrane surrounding cells. Lipids have a hydrophilic head-group bonded to long hydrocarbon ‘tails’. These lipids self-assemble into lipid bilayers in which the hydrophilic head-groups form the outer and inner surfaces of the bilayer and the hydrocarbon tails form the interior of the bilayer. It is important for cellular function that the lipid bilayer surrounding the cell incorporates lipids whose fatty acid tails have the correct stereochemistry. The two fatty acids shown in Figure 9B, oleic acid and elaidic acid, differ only in the configuration of the double bond, however, only oleic acid is found in cell membranes. The physical properties of a cell membrane are dependent on how well the lipid tails pack together. If the tails pack together very tightly, as in a bilayer containing only saturated fatty acids, then the membrane becomes rigid and does not function properly. Incorporation of fatty acids with *cis-*double bonds creates disorder among the lipid tails which leads to a more flexible cell membrane. Organisms that are adapted to low temperature have a higher proportion of unsaturated *cis-*fatty acids than those living at moderate temperatures; this helps to prevent the lipid freezing at low temperatures (for more details, see [4]).

In some systems, it is the interconversion between *cis* and *trans* isomers that is important for biological function. This is the case for retinal in the opsin proteins of photoreceptor cells (Figure 9C). In the ground state, retinal adopts the 11-*cis* configuration. On absorption of a photon of light in the visible range, isomerization to all-*trans*-retinal takes place. The changes in the shape of retinal due to isomerization trigger changes in the 3D structure of the opsin protein, ultimately leading to a nerve impulse to the brain where the visual signal is interpreted.

#### Optical isomerism and chirality

A carbon atom bonded to four different substituents is called an asymmetric carbon atom or chiral centre, as there is no centre or plane of symmetry associated with it. There are two possible spatial arrangements for the four substituents around the central carbon; these two arrangements are mirror images. If there is just one asymmetric carbon atom in a molecule, then there are two possible stereoisomers, known as enantiomers. The physical properties of the enantiomers are identical except that they rotate plane polarized light in opposite directions; for this reason enantiomers are sometimes called optical isomers. The enantiomer that rotates polarized light clockwise is designated (+) and that which rotates polarized light anticlockwise is designated (–).

**Figure 10:**
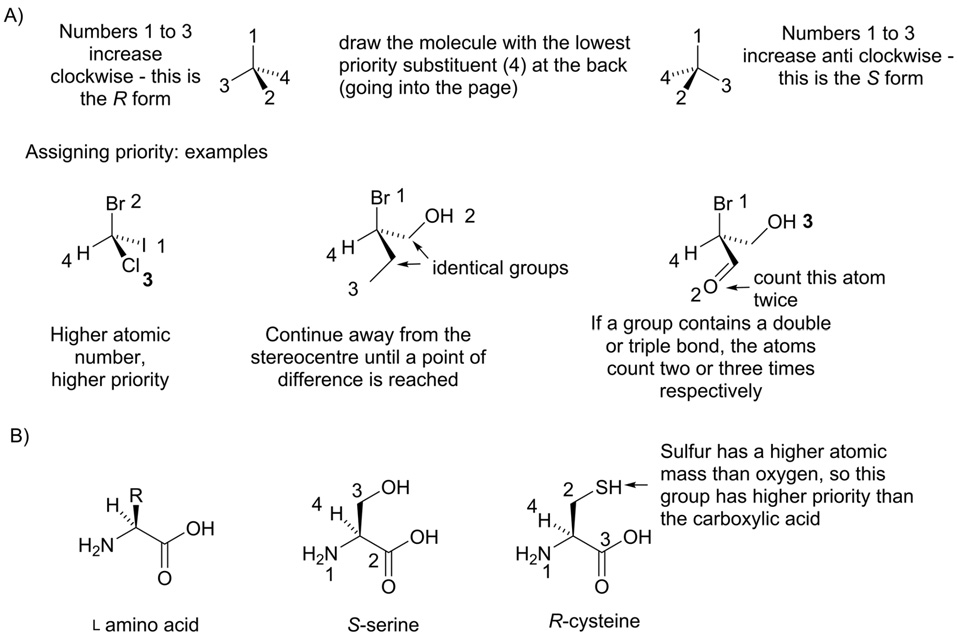
Optical isomerism (**A**) Illustration of the application of the Cahn–Ingold–Prelog rules for describing the configuration of chiral centres. In the Cahn–Ingold–Prelog system, the four substituents around the asymmetric carbon atom are assigned a priority based on three rules: (i) consider the first atom in each substituent, highest priority is given to the atom with the highest atomic number; in case of isotopes, a higher atomic mass gives a higher priority. (ii) If two substituents have the same first atom, move away from the asymmetric carbon to the next bonded atom until a difference is reached. (iii) If the substituent contains a double or triple bond, then the atom farthest from the asymmetric carbon counts two or three times respectively. The molecule is then drawn with the lowest priority substituent at the back (into the page). If the priority of the other three substituents increases clockwise, the centre is assigned ‘*R*’ stereochemistry, otherwise it is ‘*S*”. (**B**) All natural proteinogenic amino acids are the L-form under the Fischer rules, when using the Cahn–Ingold–Prelog rules the natural form of cysteine is the *R* isomer, this is in contrast with the other amino acids, exemplified by serine, which is the *S* isomer.

The ability to rotate plane polarized light is a property of the whole molecule and we cannot predict from knowledge of the structure how the plane of polarized light will rotate. Consequently, a more systematic nomenclature is desirable. Organic chemists use the Cahn–Ingold–Prelog rules to assign asymmetric carbon atoms as either ‘*R*’ or ‘*S*’ based on the arrangement of the substituents in space (Figure 10A). This system has the advantage that it can be applied to molecules with more than one asymmetric carbon atom, with each asymmetric carbon being labelled independently, and it is directly related to chemical structure. It is important to note that in molecules with more than one asymmetric carbon, not all the possible stereoisomers are enantiomers. Consider a molecule with two *R* asymmetric carbon atoms. In the enantiomer (the mirror image), these two asymmetric carbon atoms will both be *S*. However, there are two other stereoisomers in which only one of the two asymmetric carbon atoms is *S*; these two molecules are not mirror images of the first. Stereoisomers that are not mirror images of each other are referred to as diastereomers.

In biochemistry the ‘d’ and ‘l’ nomenclature, originally developed by the German chemist Emil Fischer, is still widely used to describe amino acids and sugars. In this system, the assignment is made by comparing the structure of a molecule to glyceraldehyde. In the Fisher notation, this makes all natural amino acids ‘l’, however in the Cahn–Ingold–Prelog system the naturally occurring isomer of cysteine is *R*-cysteine, while all others are the *S*-form (Figure 10B).

**Figure 11:**
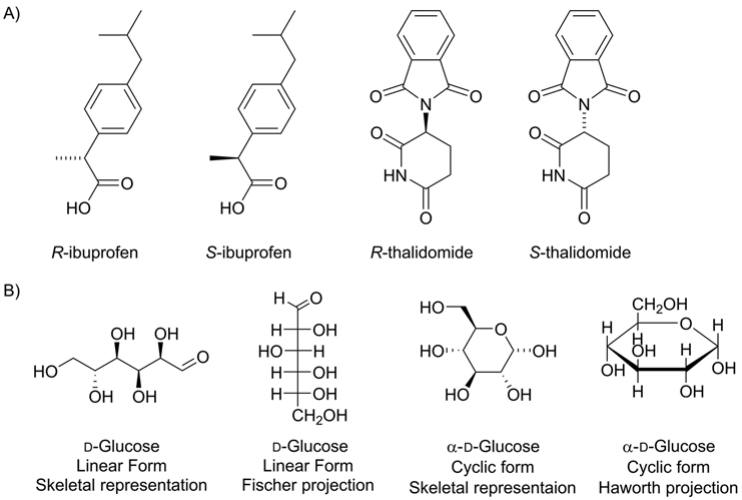
Chiral drugs and sugars (**A**) The drugs ibuprofen and thalidomide both exist as *R* and *S* isomers. In each case, the *R* and *S* isomers can be interconverted in the body and have different biological functions. (**B**) Illustration of the different representations of the glucose molecule. The linear form of glucose can be represented by a skeletal structure or a Fischer projection; the cyclic form, by a skeletal structure or a Haworth projection.

In a biological chemical reaction, an enzyme must be able to specifically recognize its substrate, and this occurs through matching of the 3D structure of the substrate to that of the enzyme active site (for more detail on active sites see [5]). For this reason, the chirality of a molecule has a huge influence on its biological activity. Typically, an enzyme will catalyse a reaction with only one of a pair of enantiomer; the other enantiomer has the wrong shape to make favourable non-covalent interactions with the enzyme-binding site. This is often important in drug design as many drugs are chiral molecules. For example, ibuprofen exists as both *R* and *S* isomers (Figure 11A). Only the *S*-form is active, however the body converts the *R*-form into the *S*-form and so the mixture of both enantiomers is effective in pain relief [6]. Similarly, the drug thalidomide (Figure 11A) exists as both *R* and *S* isomers which can be interconverted in the body. In this case, however, while the *R*-form is an effective sedative the *S*-form causes severe birth defects.

#### Specialist representations for molecules with many chiral centres

Although the majority of organic molecules relevant to biochemistry are drawn using the skeletal representation, there are some classes of molecules for which other representations are favoured. As we will discuss in more detail later, sugars can exist either in a straight chain or ring form. The straight chain form of a sugar is often depicted as a Fischer projection, while the ring form is often shown using a Haworth projection. Many different stereoisomers of sugars occur in nature, and the Fischer and Haworth projections were developed to make it easy to distinguish between stereoisomers. For example, the different representations of d-glucose (C_6_H_12_O_6_) are shown in Figure 11B.

## Why do chemical reactions happen?

### Thermodynamics

Thermodynamics aims to understand whether or not a chemical reaction will happen. In answering this question, three quantities are commonly considered: enthalpy, *H*; entropy, *S*; and the Gibbs free energy, often simply referred to as free energy, *G*. Enthalpy is a measure of the total heat content of a system. For a chemical reaction the change in enthalpy, *ΔH*, is related to factors such the number of bonds that are made and/or broken. When *ΔH* for a chemical reaction is negative, heat is given out and the reaction is called exothermic; conversely when *ΔH* is positive the system absorbs heat and the reaction is termed endothermic. Entropy is a measure of the disorder of a system; the more disordered the system the higher its entropy. The change in entropy for a chemical reaction, *ΔS*, will be positive if the disorder increases, for example when ice melts allowing the water molecules to move more freely. Changes in entropy govern whether a chemical reaction will occur; the second law of thermodynamics states that *the entropy of the universe always increases in a spontaneous process.* The problem with using this definition directly is that it is difficult to think about what happens to the universe as a whole when we are looking at one isolated chemical reaction; this is where the concept of free energy is useful. We consider the reaction and its solvent as the *system*. The change in the free energy of a system is defined in terms of the changes in enthalpy and entropy of the system:

ΔG=ΔH−TΔS

where, *T* is temperature in Kelvin. The free energy of the system is related to the entropy of the universe by the equation:

$$\Delta S_{universe}= -\frac{\Delta G}{T}$$

It can therefore be seen that in a spontaneous process the free energy of a system will decrease, i.e. *ΔG* must be negative. It is important to note that in chemical terms, spontaneous means ‘thermodynamically allowed’; a thermodynamically allowed reaction might not take place if, for example the energy barrier to the reaction (see *Kinetics* section) is too high.

The actual free energy change that takes place when reactants are mixed together depends upon physical and environmental conditions such as the concentration and physical state of reactants and the pressure and temperature of the system. Therefore, in order to be able to compare the magnitude of free energy changes for different reactions, we define a set of standard conditions; the standard symbol ‘°’ is used to indicate this. The standard symbol ‘°’ implies that all species present in the reaction are in their *standard states*: gases at a pressure of 1 bar; pure solid; pure liquid; solutions at a concentration of 1 mol dm^−3^. Importantly, the standard symbol does not imply any particular temperature. As the magnitude of a free energy change depends upon temperature it is important to state the temperature at which the value of the standard change applies.

The standard free energy change for a chemical reaction, Δ*G*°, is the change in free energy for reactants combined in molar stoichiometric amounts according to a balanced chemical equation if the reactants were converted completely into products. Consider, for example the formation of water from hydrogen and oxygen:

$${\text{H}}_{2\;}(g) +\frac{1}{2}{\text{O}}_{2}\;(g) \to {\text{H}}_{2}\text{O }(l)$$

The standard free energy change is for one mole of hydrogen gas reacting with half a mole of oxygen gas to form one mole of liquid water, with all species present in their standard state under standard conditions.

#### Chemical equilibrium

Standard free energy changes refer to reactions that proceed to completion and take place under conditions that are far removed from those experienced in biological systems, where reactions typically involve micromolar rather than molar quantities. What relevance do standard free energy changes have for biology? Standard free energy changes are enormously useful because they can be used to predict to what extent a reaction will take place in a *chemical equilibrium*.

All reactions are reversible; if we the consider the system:

A + B ⇌ C + D

The ⇌ sign indicates that both the forward reaction (A + B combining to form C + D) and the reverse reaction (C + D combining to form A + B) occur. Although, all reactions are theoretically reversible, under certain conditions either the forward or reverse reaction may predominate to the extent that the other reaction can be neglected. Reversible reactions proceed until the concentrations of the species present cease to change with time; it is said that the system has reached *chemical equilibrium*. Importantly, at equilibrium the concentrations of reactants and products are constant not because the forward and reverse reactions have stopped (static equilibrium) but because the rate of the forward reaction is equal to the rate of the reverse reaction (dynamic equilibrium). The *equilibrium constant, K*, (often referred to as *K*c in schools) is related to the concentrations of species at equilibrium and is constant for a reaction at a given temperature. In general, for the reaction:

$$a\text{A }+b\text{B }⇌c\text{C }+d\text{D }\;K=\frac{{\left[C\right]}^{c}{\left[D\right]}^{d}}{{\left[A\right]}^{a}\;{\left[B\right]}^{b}}$$

The values for *K* can vary widely from reaction to reaction. If *K* has a large value (i.e. *K*>>1), then at equilibrium the concentration of products C and D will be larger relative to the concentrations of reactants A and B, and the reaction is said to be *product favoured* or the *equilibrium lies far to the right.* If *K* has a small value (i.e. *K*<<1), then at equilibrium the concentrations of reactants A and B will be larger relative to the concentrations of products; this type of reaction is said to be *reactant favoured* or the *equilibrium lies far to the left*.

The equilibrium constant for a chemical reaction is related to the standard free energy change for the reaction, Δ_r_*G*°, by the equation:

$$\Delta_{r}G^{\text{o}}=-RT\ln K$$

where, *R* is the molar gas constant: 8.314 J mol K^−1^, *T* is the temperature in Kelvin, *ln* is the natural logarithm.

Rearranging this equation to make *K* the subject gives:

$$K=exp\;\left(\frac{-\Delta_{r}G^{\text{o}}}{RT}\right)$$

Thus, Δ_r_*G*° tells us the value of the equilibrium constant and, in turn, how far the reaction lies to the left or the right. For example, if Δ_r_*G*° is large and negative, *K* will be a large positive number and the reaction will favour the products.

#### Equilibrium in living systems; flux and coupled reactions

In biological systems, reactions do not occur in isolation but are a part of complex and interconnected metabolic pathways. In a metabolic pathway, the product of one reaction is immediately used as a reactant in the next; this creates a state of flux. In such a pathway, the concentrations of products and reactants are constantly changing. For some reactions in the pathway, the concentrations of reactants and products are close to the equilibrium values, while for many reactions the concentrations lie very far from their equilibrium values. Reactions in a metabolic pathway that are far from equilibrium can be important points for control of metabolic flux within the pathway.

Many biochemical reactions, such as formation of the bond between two amino acids in a protein, are unfavourable in isolation. For example, the standard free energy change for the formation of an amide bond in the simplest dipeptide glycine–glycine at 37°C is 15 kJ mol^−1^; and the associated equilibrium constant is 0.00297 [7], thus the equilibrium strongly favours the reactants. In order to drive the reaction forward, biochemical systems often couple reactions with a positive free energy change to those with a large negative free energy change. One reaction with a large negative free energy change that is very commonly used is the hydrolysis of ATP, which has a standard free energy change of –30 kJ mol^−1^ at 37°C. When the hydrolysis of ATP is coupled to the formation of an amide bond, the free energy change for the system becomes –15 kJ mol^−1^, and so we would expect the coupled reaction to favour the products. Importantly, coupling reactions together does not involve just having the two reactions happen together at the same time; the free energy released through ATP hydrolysis has to be captured and used to drive forward the other reaction. This capture can be, for example through causing a conformational change in an enzyme or through direct modification of the chemicals involved in the unfavourable reaction (see section on *Nucleophilic substitution at a carbonyl*).

### Kinetics

Thermodynamics refers to whether or not a reaction will be spontaneous; kinetics refers to whether a reaction will occur at an appreciable rate. In a chemical reaction, existing bonds break while new ones are formed. The transient species that occur along the reaction pathway are high in energy; the point at which the energy is highest is called the *transition state* or *activated complex*. The energy which is required in order to form the transition state is called the *activation energy*. The activation energy may be thought of as a barrier which must be overcome by reacting molecules as they collide in order for the reaction to proceed to the formation of products.

In a chemistry laboratory, the rate of a chemical reaction may be changed by altering certain properties of the system. Increasing the concentrations of the reactants (e.g. reducing particle size for solids or increasing the pressure for reactions involving gases) raises the probability of a collision between reactants and hence increases the rate of reaction. By increasing the temperature, the kinetic energy of the reactant molecules is increased. As a result, collisions between reactant molecules are more energetic such that the activated complex is more likely to form. Unfortunately, these changes are not usually possible in biology.

**Figure 12:**
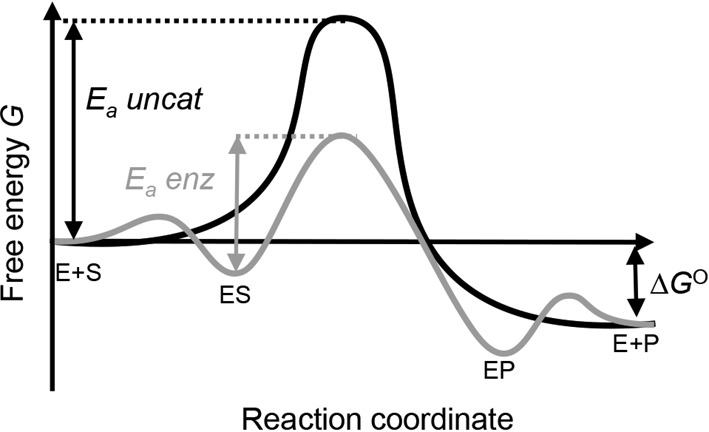
Reaction co-ordinate for an uncatalysed compared with enzyme-catalysed reaction A hypothetical energy profile for the reaction of a substrate (S) forming product (P) in the absence of catalysis is shown in black, while the hypothetical profile for the same reaction in an enzyme (E) catalysed reaction is shown in grey. In the absence of an enzyme, there is a large activation energy (*E*_a_*uncat*). In the enzyme-catalysed reaction the enzyme forms a complex with the substrate (ES) and the product (EP) and lowers the energy of the highest energy species on the reaction pathway, resulting in a lower activation energy (*E*_a_*enz*).

Addition of a catalyst can also enhance the rate of a chemical reaction. In this case, the catalyst provides an alternative route/mechanism for the reaction that involves a lower activation energy (Figure 12). Importantly, the activation energy for the reverse reaction is also lowered by a catalyst. Thus, a catalyst speeds up the rate at which the equilibrium between reactants and products can be attained but the position of the equilibrium is not changed by the catalyst. Catalysis is essential for biological reactions as the concentrations of reactants in living systems are generally very small (often micromolar to millimolar range), and the temperature at which the majority of such systems operate is relatively low such that the rates of reaction would be extremely slow without any other influences. Enzymes are very efficient catalysts which ensure that the chemical reactions involved in metabolism proceed at a useful rate. For further discussion on enzyme catalysis, see [5].

## Acids and bases

An acid is an H^+^ ion or proton donor, while a base is an H^+^ ion acceptor. Understanding which biological molecules are acids and which are bases can be important in predicting a variety of behaviours in biological systems, for example in determining catalytic mechanisms. Acids and bases can be strong or weak. When a strong acid or base is added to water, these species are almost completely ionized (dissociated). In contrast, a weak acid or base is only partially ionized on addition to water. Weak acids and bases are more relevant in biochemistry.

Consider ethanoic acid, more commonly called by its non-IUPAC name acetic acid, which is only partially dissociated in aqueous solution:

$${\text{CH}}_{3}\text{COOH }(\text{aq}) ⇌{\text{H}}^{+}\;(\text{aq}) +{\text{CH}}_{3}{\text{COO}}^{-}\;(\text{aq})$$

For acetic acid, the equilibrium lies well to the left such that the concentration of undissociated acetic acid (CH_3_COOH) is much greater than that of H^+^ ions or acetate ions (CH_3_COO^−^). The equilibrium constant for this reaction indicates the extent to which the dissociation occurs; the equilibrium constant for acid dissociation reactions is often called the *acid-dissociation constant, K*_a_*.* Considering a general acid as HA:

$$\text{HA }⇌{\text{H}}^{+}\;+{\text{A}}^{-}\;K_{a}=\frac{\left[H^{+}] [A^{-}\right]}{HA}$$

The acid-dissociation constant for acetic acid at 25°C is 1.76 × 10^−5^, therefore a 0.1 mol dm^−3^ solution of acetic acid is only 1.3% ionized. The species remaining after a proton has dissociated from an acid is referred to as its conjugate base. For example, the acetate ion is the conjugate base of acetic acid; acetic acid and acetate are referred to as a conjugate pair.

Acetic acid is one of the many acids containing the –COOH or carboxylic acid functional group. Many carboxylic acids are present in biological systems and importantly commonly serve as H^+^ donors during enzymatic catalysis. Similarly, the conjugate base of the carboxylate ion can act as a proton acceptor in base-catalysed enzyme reactions.

### pH and p*K*_a_

The concentration of H^+^ in aqueous systems is typically very low. For example, the concentration of H^+^ in pure water at 25°C is 10^−7^ mol dm^−3^. The pH scale is a convenient way of representing very small concentrations; by definition:

$$\text{pH }= -\log_{10}[{\text{H}}^{+}\text{]}$$

Acid dissociation constants have a very large range and analogous to pH, the quantity p*K_a_* is defined:

$$\text{p}K_{a}=-\log_{10\;}K_{a}$$

One reason for using *pK_a_* is that this makes it easy to work out to what extent an acidic species will be protonated at a given pH. The relation between pH and p*K*_a_ is known as the Henderson–Hasselbalch equation:

$$\text{pH }=\text{p}K_{a}+\log_{10\;}([{\text{A}}^{-}]/[\text{HA}])$$

where, HA is an acid and A^–^ is its conjugate base. Without considering specific concentrations, using this equation we can see that if the pH of a solution is equal to the p*K*_a_ of the acid, then the concentration of the acid form will equal that of the conjugate base. Similarly, at a pH considerably below the p*K*_a_ the acid form, HA, will dominate, while at a pH considerably above p*K*_a_ the conjugate base, A^−^, will dominate.

Several amino acids found in proteins have ionizable side chains; knowledge of typical p*K*_a_s of the functional groups in these amino acids allows us to predict the ionization state at physiological pH. In a protein, the side chain of aspartic acid, for example typically has a p*K*_a_ of ~3.5; at physiological pH, we would expect this group to be ionized/deprotonated.

Interestingly, although we can use typical p*K*_a_ values as a crude approximate of how a particular amino acid side chain will behave in a protein, in some cases the p*K*_a_ of that side chain can vary wildly from the typical value. This is because the 3D structure of a protein can create a local environment in which a proton can be lost or gained more readily. Consider the aspartate side chain mentioned above; if there is a positively charged amino acid side chain in the vicinity, then the deprotonated state with its negative charge will form more readily. The majority of the p*K*_a_ values for aspartic acid side chains in proteins reported in the literature are within ±1 of the average value. The lowest reported p*K*_a_ is however 0.5 and the highest 9.2 [8].

### Buffers

A buffer is a solution that maintains a relatively constant pH even upon addition of an acid or a base. Buffers typically consist of a weak acid and a salt of its conjugate base. The weak acid or conjugate base can absorb additions of OH^−^ or H^+^ allowing the pH of the solution to remain approximately constant. Consider the pH of 1 mol dm^−3^ buffered solution containing 0.25 moles of acetic acid and 0.25 moles of acetate. Since the solution contains equal amounts of a weak acid and its conjugate base, the pH is equal to the p*K*_a_ of the weak acid, 4.75 in this case. When 0.05 moles of a strong acid is added to the buffer, the H^+^ from the strong acid will react with the acetate ion in the buffer, creating more acetic acid and water. This will cause the total amount of acetate to decrease to 0.20 moles and the total amount of acetic acid to increase to 0.30 moles. Using the Henderson–Hasselbalch equation, we find that the new pH of the solution is 4.57, a change in just 0.18 pH units when 0.05 moles of strong acid is added to the buffer. In contrast, adding the same 0.05 moles of strong acid to 1 l of pure water causes the pH to change from 7.00 to 1.32, a change of 5.68 pH units.

A buffer has its greatest pH buffering capacity when the concentration of the weak acid is equal to the concentration of its conjugate base, and when the desired pH of the solution is within one pH unit of the p*K*_a_ of the weak acid. For example, consider making an acetate buffer with pH of 4.50. As the desired pH is below the p*K*_a_ of acetic acid (4.75), the Henderson–Hasselbalch equation predicts that we will need 1.78 times as much acetic acid as acetate ion in the solution. This solution will still have large amounts of both acetic acid and acetate in solution to react with added acid or base to keep the pH of the solution stable. However, if the desired pH of solution is 7.00, then the calculated ratio of acetic acid to acetate is 0.0056, meaning there would be practically no acetate ions in the solution. If a strong acid was added to this solution, the pH would change dramatically.

**Figure 13:**
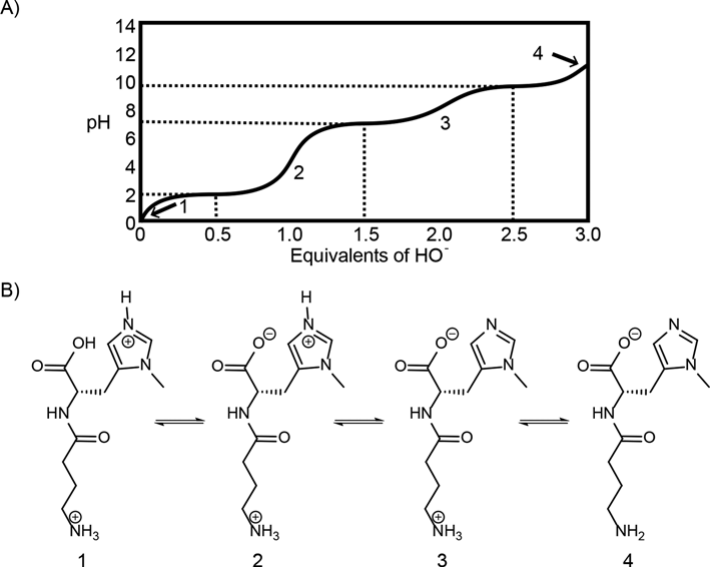
Anserine buffering (**A**) A schematic titration curve for anserine, with (**B**) an illustration of the species present in the reaction mixture during the titration. Anserine has three groups that can exist in both protonated and unprotonated forms: a carboxylic acid, an imidazole nitrogen and an amine. At the beginning of the titration, all the three groups are protonated and the predominant species in the reaction mix is (1). As base is added, the pH approaches the p*K*_a_ for the first ionizable group, the carboxylic acid, and species (2) begins to form resulting in a plateau in the titration curve. Once the majority of molecules are in form (2), the pH will begin to rise again, before the second plateau occurs due to the formation of species (3). It is the equilibrium between species (2) and (3) that is relevant at biological pH. The final plateau occurs when the fully deprotonated species (4) is formed.

Maintaining a constant pH is vital to many biological processes. Metabolic pathways depend upon the catalytic activity of enzymes that contain many ionizable functional groups. As described above, changing the pH can alter the protonation state of functional groups in the active site of the enzyme altering its activity. One important buffering system for maintaining intracellular pH, typically between 6.9 and 7.4 for most cells, involves protonation of a modified histidine side chain. Free histidine itself is not a good candidate for an intracellular buffer as the concentration of free histidine in cells is typically low and the p*K*_a_ of its imidazole group (6.0) is greater than one pH unit away from the typical pH range in a cell. However, in the dipeptide anserine, the p*K*_a_ of the imidazole nitrogen in the side chain of the methyl histidine is raised to 7.0 making it an ideal candidate for an intracellular buffer (Figure 13).

## Reactions in organic chemistry

Enzymes enhance the rate of a chemical reaction by providing an environment in which intermediate species along the reaction pathway are stabilized, however, the mechanisms by which chemical reactions occur obey the same principles as those performed in a chemistry lab. We can therefore learn a great deal about a biochemical reactions by studying the mechanisms of the reactions in the absence of an enzyme. Here, we outline some of the most commonly occurring reactions in biological chemistry.

### Nucleophilic substitution reactions at saturated carbon centres

**Figure 14:**
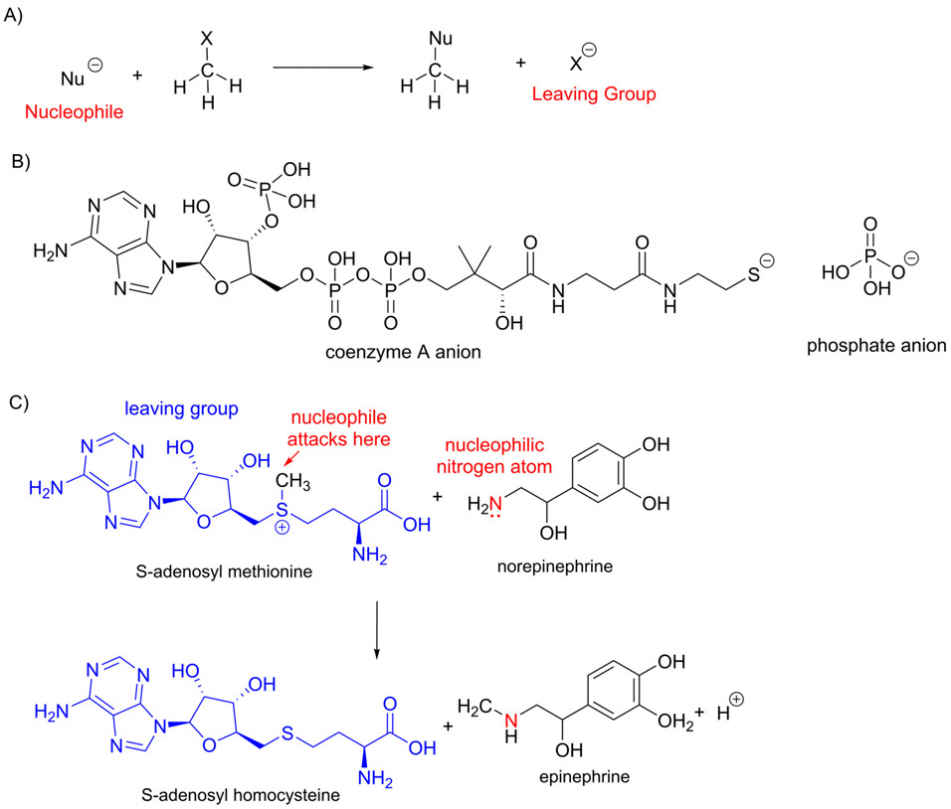
Nucleophilic substitution at a saturated carbon centre (**A**) Illustration of a nucleophilic substitution with a negatively charged nucleophile and a leaving group. It should be noted that the nucleophile may be a neutral molecule with a lone pair of electrons and that the leaving group may also be neutral (as seen in (**C**)). (**B**) Common leaving groups in biological chemistry. (C) Many biological methylation reactions take place via a nucleophilic attack on *S*-adenosyl methionine. In this diagram, the leaving group is shown in blue and the nucleophile in red.

Carbon atoms forming one or more single bonds to electronegative atoms are susceptible to attack from electron-rich nucleophiles. In the final product of a *nucleophilic substitution reaction*, the nucleophile forms a covalent bond with the carbon atom and replaces a ‘leaving group’ (often designated by the symbol X) (Figure 14A). Whether or not a nucleophilic substitution reaction will take place depends, in part, on whether ‘X’ is a good leaving group, but what is meant by a good leaving group? When the bond between the carbon atom and X breaks, it does so heterolytically, with both the electrons in the bond being transferred to X. A good leaving group is therefore one that can readily accommodate these electrons. In many cases, the leaving group will become negatively charged, so good leaving groups are often those that will form stable anions.

The coenzyme A anion, with a negative charge on sulphur and the phosphate anion, with the negative charge delocalized over several atoms, are very common leaving groups in biological chemical reactions (Figure 14B). One of the most widely occurring nucleophilic substitution reactions in biochemistry is transfer of a methyl group from *S*-adenosyl methionine to a nucleophile (Figure 14C). Many different nucleophiles are reactive towards this molecule. For example, in the synthesis of the amino acid methionine the nucleophile is a sulphur atom; in the synthesis of norepinephrine the nucleophile is a nitrogen atom; and the degradation of dopamine includes nucleophilic attack on *S*-adenosyl methionine by an oxygen atom. In each case, the *S*-adenosyl methionine is a positively charged substrate and the leaving group is the neutral *S*-adenosyl homocysteine group.

### Carbonyl chemistry

**Figure 15:**
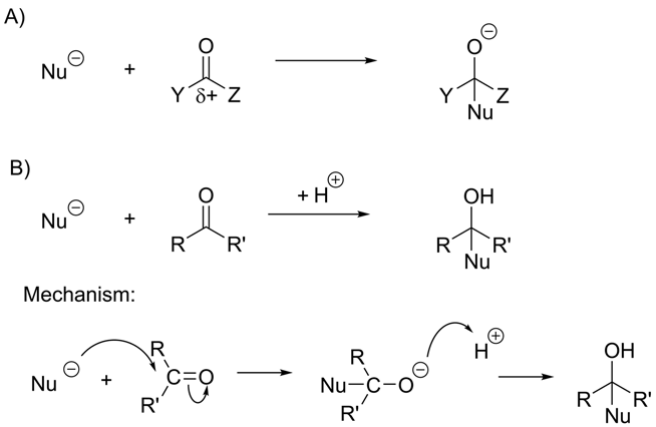
Nucleophilic addition at a carbonyl group (**A**) Nucleophilic attack on a carbonyl group results in a species with four substituents around a central carbon atom (a tetrahedral species). Whether or not this species reacts further depends upon the nature of the X and Y groups and the nucleophile. (**B**) If X and Y are not good leaving groups and the nucleophile is also a poor leaving group, then the tetrahedral species will be the final product.

The carbonyl group plays a central role in many biological chemical reactions, in particular those involving the making and breaking of carbon–carbon bonds. As mentioned earlier, the carbonyl carbon atom carries a partial positive charge, making it susceptible to nucleophilic attack by electron-rich species. The first step in the reaction of a nucleophile with a carbonyl carbon atom is the formation of a new covalent bond, resulting in a species with four substituents around the central carbon atom (Figure 15A). What happens after this initial attack depends on which other atoms are bonded to the carbonyl carbon atom in the starting material. One possibility is that the negatively charged oxygen atom will form a bond to a proton to form an alcohol, and the central carbon atom will have four substituents in the final product. Another possibility is that the carbonyl group will reform, displacing one of the other three substituents. A final possibility is that the attacking nucleophile is itself a good leaving group, and so the reaction reverses. Which of these possibilities occur depend on whether the carbonyl group in the starting material is bonded to a good leaving group, the reactivity of the nucleophile and on the relative thermodynamic stability of the starting materials and products.

#### Nucleophilic addition to a carbonyl

**Figure 16:**
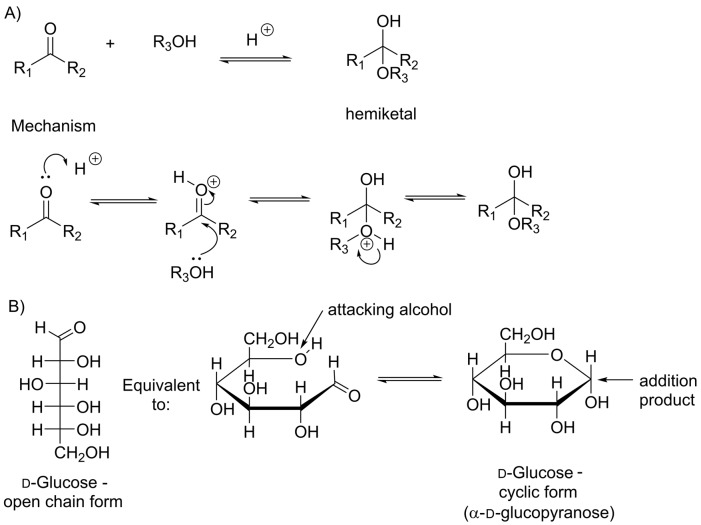
Reversible addition at a carbonyl group (**A**) If X and Y are not good leaving groups but the nucleophile is a good leaving group, then the reaction may reverse, as seen in the acid-catalysed reaction of a ketone with an alcohol. This reaction also occurs with aldehydes and the tetrahedral product is called a hemiacetal. (**B**) Formation of hemiacetal occurs during the cyclization of glucose.

When a nucleophile reacts with an aldehyde or a ketone, there are two possible outcomes: addition of the nucleophile or reversal of the reaction to regenerate the starting material. Addition, rather than substitution, occurs because neither ‘H^–^’ nor ‘R^–^’ (with a negatively charged carbon atom) are good leaving groups (Figure 15B). An addition reaction usually involves a strong nucleophile and can be very useful in forming new carbon–carbon bonds. The reverse reaction occurs when the nucleophile is itself a good leaving group, for example water or an alcohol. This usually results in an equilibrium between the carbonyl compound and the addition product. When this reaction is carried out in the organic chemistry laboratory, an acid catalyst is used to enhance the reactivity of the carbonyl group (Figure 16A). The addition of an alcohol to an aldehyde or ketone is responsible for the cyclization of sugars. In cells, an enzyme catalyses sugar cyclization and the attack on the carbonyl group is made by an alcohol in the same molecule (Figure 16B).

#### Nucleophilic substitution at a carbonyl

**Figure 17:**
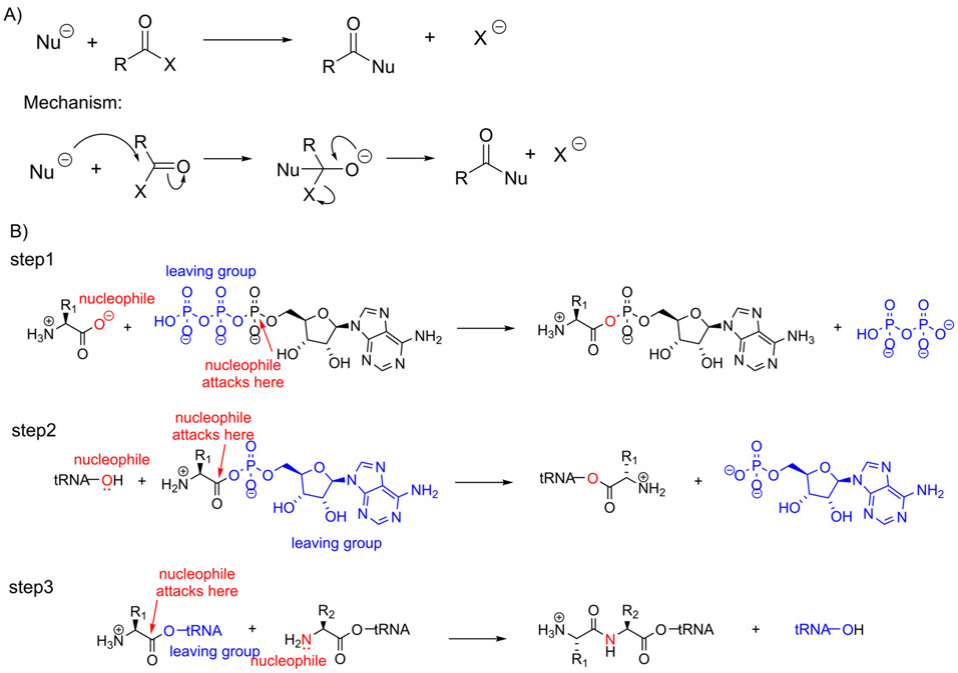
Nucleophilic substitution at a carbonyl group (**A**) If the carbonyl subjected to a nucleophilic attack has a good leaving group, then nucleophilic substitution is possible. The substitution reaction proceeds via an obligatory tetrahedral species. (**B**) Three nucleophilic substitution reactions are important in the formation of an amide bond between two amino acids. The first reaction involves nucleophilic substitution at a phosphate group and the second nucleophilic reactions involve substitution at a carbonyl. In the first reaction, the carboxylate group attacks a phosphate of ATP releasing pyrophosphate – two phosphate groups connected together via a bridging oxygen atom – and attaching AMP to the carboxylate group. This step is energetically favourable and converts one of the oxygen atoms of the carboxylate group of the amino acid into a good leaving group. In the second reaction, the amino acid is loaded on to the tRNA ready for protein formation. In the third reaction, the enzyme ensures that the amino group of the attacking amino acid is not protonated, and this group then carries out a nucleophilic attack on another tRNA–conjugated amino acid.

If the carbonyl carbon in the starting material is bonded to a good leaving group, then nucleophilic substitution reactions are a possibility. In substitution reactions, the incoming nucleophile makes a bond with the carbonyl carbon and the bond between the carbonyl carbon and the leaving group breaks. These processes do not happen simultaneously; the addition of a nucleophile to a carbonyl compound always forms a species with four substituents around the central carbon first (Figure 17A). Nucleophilic substitution at a carbonyl is the key reaction in protein synthesis, resulting in a carboxylate group being converted into an amide. The formation of the amide does not occur by nucleophilic attack of the amine of one amino acid on the carboxylate group of another, however. First, in order to make the reaction energetically favourable, it must be coupled to the hydrolysis of ATP. Second, from a mechanistic perspective, the carboxylic acid/carboxylate group does not have a good leaving group and so is a poor substrate for nucleophilic substitution. Third, the amino acid needs to be attached to a biological macromolecule (a tRNA) in order to make sure that the correct amino acid is added to the growing peptide chain. In order to meet these requirements, a peptide bond is made in a series of three reactions (Figure 17B).

#### Enol and enolate formation

**Figure 18:**
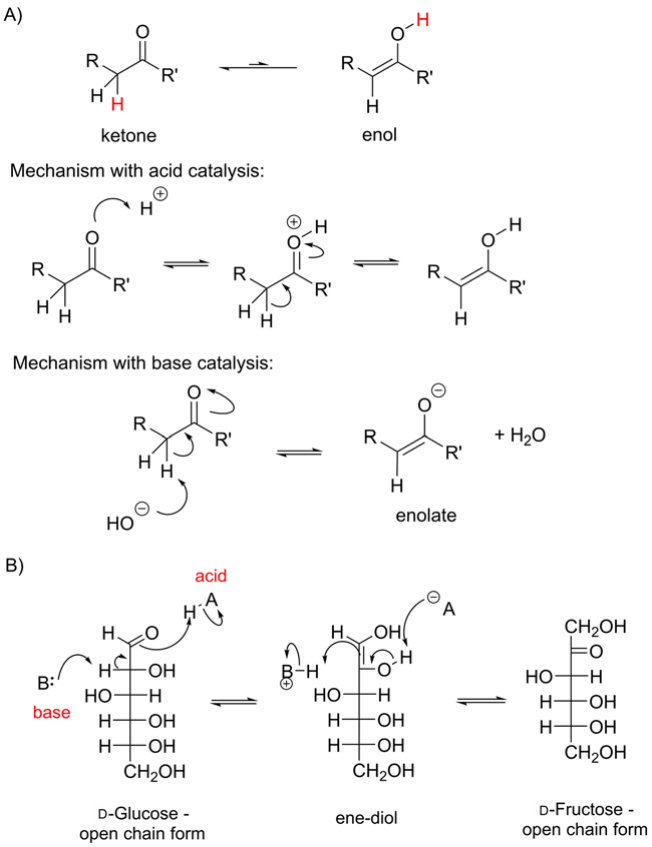
Keto–enol tautomerism (**A**) Illustration of a ketone and its enol form, and the mechanism of this reaction in the presence of acid and base. For the majority of species, the equilibrium lies towards the ketone rather than the enol. (**B**) The tautomerization of glucose gives an ene–diol, because there is an alcohol group adjacent to the carbonyl (diol, two alcohols). If, in the reverse reaction the carbonyl group is formed at the alcohol adjacent to the original carbonyl in the starting material, then an isomer of glucose, fructose, is formed.

**Figure 19:**
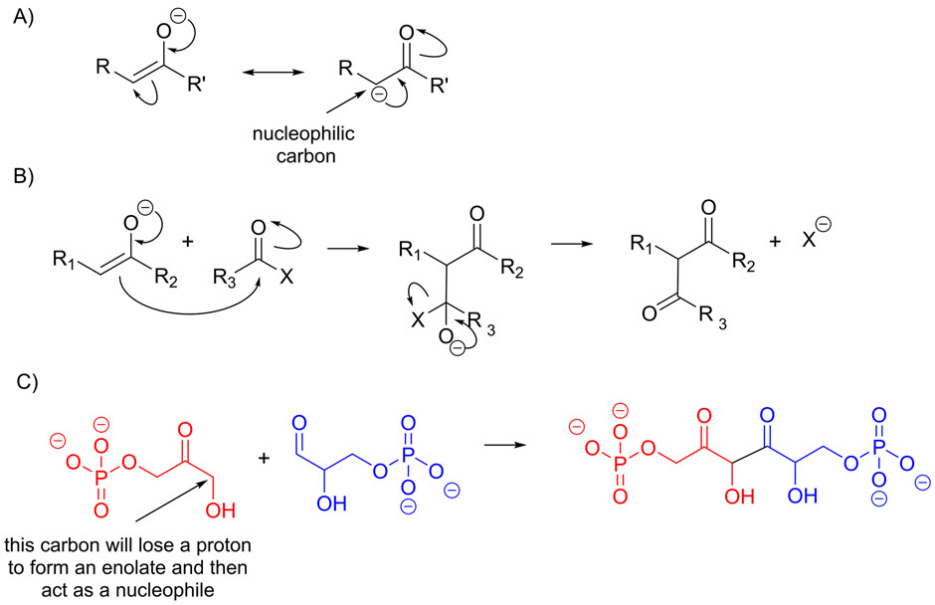
Carbon–carbon bond formation with enols/enolates (**A**) Resonance structures of an enolate illustrating that there is high electron density on both the oxygen atom and one of the carbon atoms in this molecule. (**B**) When an enolate attacks a carbonyl group through its nucleophilic carbon, a new carbon–carbon bond is formed. If there is a good leaving group, X, on the tetrahedral intermediate species then a second carbonyl group can form, otherwise the tetrahedral species can gain a proton to form an alcohol. (**C**) The reaction of dihydroxyacetone phosphate (red) and glyceraldehyde-3-phosphate (blue) to form fructose-1,6-bisphosphate is a key reaction in glucose synthesis, while the reverse reaction occurs in the breakdown of glucose (glycolysis).

Many carbonyl compounds show a form of isomerism known as tautomerism, in which a proton moves from one position in the molecule to another. In general, the equilibrium lies towards the carbonyl compound, although there are some exceptions. Enol and enolate formation can be catalysed by either acid or base *in vitro*; *in vivo* this isomerism is catalysed by an enzyme, and the amino acid side chains making up the active site act as acids and bases during the catalytic cycle (Figure 18A). In biological chemistry, keto–enol tautomerism takes place during the interconversion of different types of sugars (Figure 18B). It is also very important in the formation of new carbon–carbon bonds as enols and enolates can act as carbon nucleophiles. We can understand why this is if we consider the resonance structure of the enolate anion which has a negative charge on a carbon atom (Figure 19). Although the resonance structure does not exist as a stable molecule, it helps us to understand why this particular carbon can be a nucleophile. Although carbon with a negative charge is usually very unstable, in an enolate the bonding is actually very similar to that seen in a carboxylate – the negative charge is not localized on the carbon atom but is instead delocalized over three atoms, which has a stabilizing effect. This reaction is very important in the synthesis and degradation of both sugars and fatty acids.

### Oxidation and reduction of organic compounds

**Figure 20:**
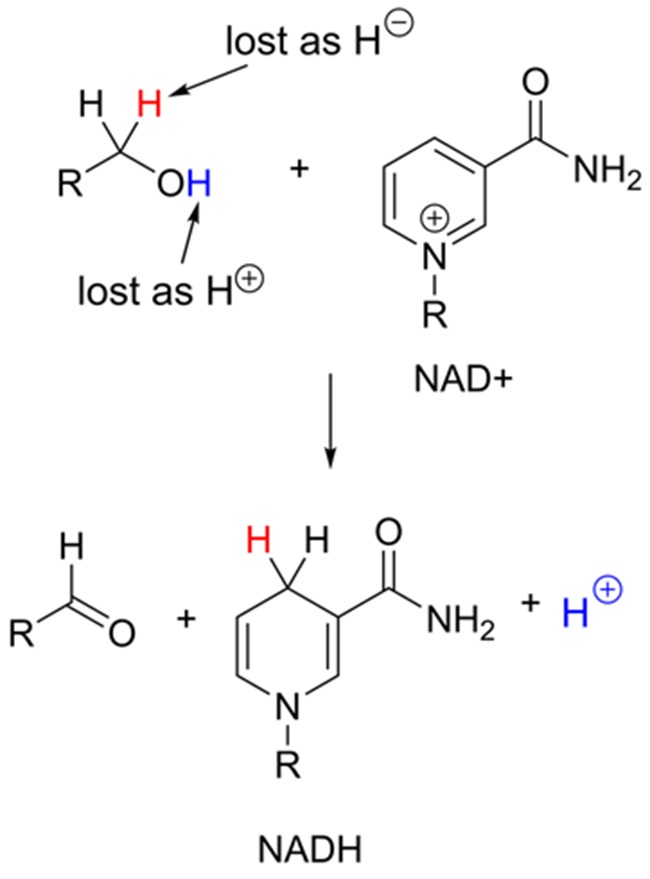
Organic reduction reactions NAD^+^ is the acceptor molecule for hydride ion in many organic oxidation reactions. It also donates hydride ion in reductions of, for example, carbonyl derivatives.

Oxidation of a compound involves loss of electrons and reduction involves gain of electrons during a chemical reaction. You will probably be most familiar with this process in inorganic chemistry, where metal ions change their oxidation state through addition or loss of electrons. Organic molecules can be oxidized or reduced, however the electrons are often transferred indirectly, for example through transfer of ions. This indirect transfer often takes the form of hydrogen atoms; in the oxidation of an alcohol to a ketone, two hydrogen atoms are lost by the alcohol, with the concomitant loss of two electrons in the process. The electrons are often passed to an acceptor molecule as a hydride ion H^–^. A hydride ion is very unstable and is a very poor leaving group, however in this case, the enzyme ensures that the acceptor molecule is perfectly positioned to receive the hydride in the enzyme active site. There are a number of different hydride acceptor molecules in biochemical systems, a very common one is NAD^+^ (Figure 20).

## Metal ions

There are some processes that are essential for life for which non-carbon-based chemistry is required. In particular, life makes use of the varied properties of metals. For example, the ability of the transition metals to exist in multiple oxidation states is often exploited in biology to aid the catalysis of oxidation reduction reactions; transition metals often act as electron carriers in these reactions.

The process of photosynthesis includes electron transfer steps utilizing transition metals. One example is the use of ferredoxins to transfer electrons from photosytem I to the protein responsible for making the reducing agent NADPH, which is necessary for reducing CO_2_ to form glucose. Ferredoxins are proteins that contain one or more clusters of iron ions and sulphide ions, and some of the iron ions in these clusters are able to cycle between the Fe^3+^ and Fe^2+^ state.

$$Fe^{3+}+e^{-}↔Fe^{2+}$$

In photosynthesis, the ferredoxin protein contains a cluster with two iron and two sulphur ions, designated as [2Fe2S]. In the oxidized state, both the iron ions are Fe^3+^. When ferredoxin docks with reduced photosystem I, one of the iron ions receive an electron and become Fe^2+^. The ferredoxin diffuses to the next protein in the chain (ferredoxin:NADP^+^ reductase), where the Fe^2+^ acts as an electron donor. A second example illustrating the importance of transition metals in photosynthesis is the manganese-containing cluster in photosystem II. In this case, the oxidation state of the manganese ions change; essentially acting like a biological capacitor building up positive charge, driven by light. This charge is then neutralized by taking the electrons from water, producing oxygen (for a further discussion see [9]).

The ability of metal ions to co-ordinate non-metal ligands is also used extensively within biology in order to maintain specific protein structures. For example, zinc ions are used in *zinc finger* proteins to support a structure that can recognize and bind to DNA. In addition, the variation in charge, size and preferred pattern of co-ordination among different ions allows them to be recognized by biological molecules. This in turn allows metal ions to be used as signalling molecules in biological systems, for example calcium ions in muscle contraction. In addition the solubility of metals within water provides life with both challenges and opportunities. Clearly, the levels of these metals must be carefully controlled to stop unwanted precipitation, but this process can be harnessed to enable life to produce hardened structures such as shells and bones. Overall, it is the different binding properties of metals compared with carbon, along with the ability to exist in multiple oxidation states which metal ions bring to the diversity of chemistry in life.

## Concluding remarks

Life carries out an astonishing variety of chemistry under mild conditions and with enormous efficiency. Here, we have provided a brief overview of the essential principles that govern the reactions of life. We hope that interested readers will refer to some of the suggested resources which provide more detail on why chemical reactions occur.

This article is a reviewed, revised and updated version of the following ‘Biochemistry Across the School Curriculum’ (BASC) booklet: *Essential Chemistry for Biochemistry* by E.J. Wood and A. Myers (1991). For further information and to provide feedback on this or any other Biochemical Society education resource, please contact education@biochemistry.org. For further information on other Biochemical Society publications, please visit www.biochemistry.org/publications.
